# Establishing a linked administrative data sampling frame to recruit families into a new UK-wide birth cohort study: findings from the Early Life Cohort Feasibility (ELC-FS) Study

**DOI:** 10.23889/ijpds.v9i5.2749

**Published:** 2024-09-18

**Authors:** Orla McBride, Lisa Calderwood, Karen Dennison, Erica Wong, Alyce Raybould, Richard Silverwood, Pia Hardelid, Lucy Griffiths, Pasco Fearon, Alissa Goodman

**Affiliations:** 1 Ulster University; 2 University College London; 3 University of Cambridge

## Objective and Approach

Funded by the Economic and Social Research Council (2021-2024), a core aim of the ELC-FS was to establish a sampling frame by linking birth registrations and maternity records in the four UK nations (England, Wales, Scotland and Northern Ireland) to facilitate recruitment of ~2000 families into a new birth cohort study. An opt-out recruitment approach was essential. Widespread public dialogue work was undertaken to glean the attitudes and perspectives of stakeholders including data controllers, data users, and parents of young children, as to the acceptability of this recruitment model.

## Results

Achieving the sampling frame required nation-specific approaches due to differences in existing legislation governing secondary use of administrative data. Complex and protracted negotiations with data controller approval panels were necessary. Public dialogue work revealed that stakeholders were: (i) excited by the prospect of a new UK-wide birth cohort study to provide invaluable insights into the lives of a new generation; and (ii) broadly accepting of the proposed use of administrative data for generating a sampling frame, particularly in terms of ensuring that the ELC-FS study has diverse and inclusive representation of family life in the UK today.

## Conclusions

ELC-FS fieldwork will be completed by mid-2024. Preliminary analyses to be presented at the conference will assess the suitability of the sampling frame for achieving the target ELC-FS sample.

## Implications

With careful engagement with stakeholders at a national level, access to linked administrative data as a sampling frame for a UK-wide birth cohort study is achievable, but not straightforward.

